# Understanding young women’s experiences of gender inequality in Lucknow, Uttar Pradesh through story circles

**DOI:** 10.1080/02673843.2019.1568888

**Published:** 2019-02-15

**Authors:** Luciana E. Hebert, Suchi Bansal, Soo Young Lee, Shirley Yan, Motolani Akinola, Márquez Rhyne, Alicia Menendez, Melissa Gilliam

**Affiliations:** a Ci3, Department of Obstetrics and Gynecology, University of Chicago, Chicago, IL, USA; b Harris School of Public Policy, University of Chicago, Chicago, IL, USA

**Keywords:** Adolescents, gender, health, story circles, India, participatory methods

## Abstract

Gender inequality poses grave consequences for young women’s health and wellbeing. The aim of this study was to understand how gender influences the lives of young women living in urban slums of Lucknow, Uttar Pradesh, India using story circles as a research methodology. Narrative-based participatory methods like story circles (which involves sharing individual stories in a group circle on a given topic) can provide the nuance and detail needed to understand young people’s experiences, build trust between participants and researchers, and offer spaces to speak about culturally sensitive subjects. Six story circle sessions were conducted with 50 young women (aged 15–24) in Lucknow. Sessions were audio-recorded, transcribed, and coded. Transcriptions were analysed to identify the following salient themes, all of which act as mechanisms of gender inequality: mobility restrictions, rampant sexual harassment in the community, limited educational and work opportunities, and the utmost prioritization of marriage for young women.

## Introduction

Reducing gender inequality is a global health and development priority (United Nations General Assembly, 2015; World Health Organization, 2013). Gender inequality affects human health and wellbeing and has severe consequences for women and girls (Osmani & Sen, 2003; Sengupta, 2016; Sinha, McRoy, Berkman, & Sutherland, 2017). Systemic indicators of inequality include disparities in levels of education, economic participation, and poverty; social indicators of inequality include restrictive gender norms and gender-based violence. These systemic and social factors intersect to cause adverse health outcomes and inequalities for women. In India, where girls and women significantly trail boys and men on indicators of inequality, transforming norms and systems that reinforce gender disparities is crucial for achieving development and community health goals. Realizing gender equity requires an understanding of the context in which gender inequalities persist. Studying how gender influences the lives of adolescents can provide information about how gender inequalities are fostered and perpetuated, and provide insights into areas for intervention.

In India, gender inequalities manifest as vastly different expectations for young men and women regarding their education, health, career, and overall agency in decisions affecting their future. Uttar Pradesh (UP), India’s most populous state, has slanted sex ratios (871 young women per 1000 young men, aged 15–24 years) and a series of disparities along gender lines. For example, only 76% of young women in the region are able to read compared to 87% of young men (United Nations Population Fund, 2011). Despite laws prohibiting marriage before age 18, 21.1% of girls aged 20–24 years in UP report being married before 18 (International Institute for Population Sciences, 2017).

Adolescents’ experience with gender has long term implications for their education, economic achievement, and health (Sen, Ostlin, & Asha, 2007). Hosseinpoor et al. (2012) conducted an analysis of data on 103,154 men and 125,728 women from 57 countries in the 2002–2004 World Health Survey. This analysis revealed that women reported poorer health than men, and that 30% of this observed health inequality was ‘explained’ by age and social determinants. Employment was the largest single explanatory factor (75.5% of men reported working paid jobs, compared to only 38.5% of women), with women reporting their primary reasons for nonemployment as being homemakers and family caretakers. Educational attainment also contributes to health disparities. Higher educational attainment is associated with healthier sexual and reproductive health outcomes among women and girls, including increased contraceptive use, delayed age of marriage and first childbirth, greater uptake of prenatal care, lower infant mortality rates, and higher child vaccination rates (Chou, Liu, Grossman, & Joyce, 2010; Currie & Moretti, 2003; Mmari & Sabherwal, 2013).

Learning about young women’s personal experiences with gender, and particularly gender barriers to educational and economic achievement, is a crucial step in designing programs that support young people’s empowerment and overall health. However, cultural sensitivities around gender poses ethical and methodological challenges for research with youth on experiences around gender. Challenging areas include: gaining informed consent for a minor’s participation, building trust between researchers and youth, and having youth disclose information about their attitudes and behaviours (Flicker & Guta, 2008; Kirk, 2007; Santelli et al., 2003).

Narrative-based research methods offer benefits for research with adolescents. They provide an avenue for building trust between researchers and adolescents. Narratives can provide the nuance and detail needed to understand young people’s experiences. This kind of research mainly relies on storytelling. Storytelling is central to how one develops, reflects upon, and communicates about identity and self-concept (Larkey & Hecht, 2010; McLean, 2005), but is underused in research on adolescent sexual health and gender. One exception is a small literature on digital storytelling, a method for depicting narrative through video, narration, and music (Gubrium, 2009).

This particular study used story circles, a narrative-based research method, to explore the effect of gender on the daily life and future expectations of young women in Lucknow, Uttar Pradesh, India. Story circles require participants to listen to a prompt, then craft and narrate a story in response (Gubrium, 2009). Story circles emerged from the participatory democratic processes of the Student Nonviolent Coordinating Committee (SNCC), Free Southern Theater (FST), and Junebug Productions; they were developed and promoted in large part by John O’Neal, co-founder of FST and Junebug Productions. Unlike traditional focus group discussions, story circles use broader prompts and do not rely on probing. Instead, in a story circle, each person is given time to speak and reflect upon, share, develop, and refine their story through multiple rounds of storytelling.

The story circle methodology is a core activity in digital storytelling workshops, but there is a dearth of research focusing solely on the story circle process and data that is collected in a story circle.

## Methods

This research was conducted as part of a parent study, Kissa Kahani, which used a variety of narrative-based methods to explore the role of gender in the lives of adolescents in Uttar Pradesh, India and its relationship to sexual and reproductive health. Participants were recruited from non-governmental organizations in Lucknow. Inclusion criteria included: identifying as female, ages 15–24, and living in urban slums of Lucknow. Young women under the age of 18 gave assent to participate, while their parents gave consent; all others gave consent. The Institutional Review Board at the University of Chicago’s Biological Sciences Division and the Sigma IRB in India approved all study procedures.

### Story circle sessions

Facilitators were recruited from Indian nongovernmental organizations that use storytelling methods. Facilitators were trained over a two-week period specifically on the story circle and digital storytelling methodologies. Each story circle session included five youth and two to three female facilitators. To begin each session, facilitators introduced the story circle methodology, including a brief description of the history of story circles. Facilitators then provided a prompt, i.e. a question for reflection to which participants were invited to respond. These prompts included: *‘Tell me about a time in your life when you faced a difficulty or trouble, and how you overcame it’* and *‘Tell me about a time that your gender/being a girl influenced your future.’* Going around the circle, each participant was invited to share a story responding to the prompt. Each participant was given an equal amount of uninterrupted time to respond to the prompt, which they were allowed to interpret as they wished. The process was then repeated, allowing participants to embellish their stories after having heard stories of other participants. Following this second round of storytelling, participants were invited to respond to and reflect on one another’s stories. Upon completing multiple such rounds of storytelling and reflection, facilitators used a semi-structured guide to lead a discussion with participants, exploring commonalities and differences across narratives. After each session, the project team debriefed with facilitators, providing additional coaching on issues or ideas that arose during the session. Each story circle session was audio recorded, and each session lasted fewer than 90 minutes.

### Coding and analysis

Audio recordings of the narratives, responses, and discussion were transcribed verbatim and translated from Hindi into English by a third party. The study team verified transcripts for accuracy and later de-identified them. Four team members who were not involved in facilitation or debriefing of the story circle sessions created a preliminary codebook by reading the transcripts and identifying cross-cutting themes. Two transcripts were then coded using the preliminary codebook; as new themes emerged, they were used to guide refinement and editing of the codebook. The finalized codebook was used by two independent research team members to code transcripts. A third research team member then compared the coded transcripts for accuracy. Dedoose, an online coding and data management system, was used to organize and code transcripts.

The coding process resulted in the identification of such salient themes as: mobility, harassment, education, work, and relationships. To organize data, quotations corresponding to each theme were pulled and subsequently organized into a matrix of sub themes and representative quotations. To further analyse, summarize, and contextualize the excerpts, memos were created to synthesize quotations under a given theme and highlight patterns that emerged from the matrixing process. Other research team members not involved in initial drafting of the memos reviewed the matrices to iteratively develop and validate the emergent themes and findings. Memos went through multiple rounds of review, refinement, and reading against the quotations and against other memos to identify the most salient themes.

## Results

Six story circle sessions were conducted in February 2016 with 50 young women, resulting in 50 stories and six discussion sessions (including reflection and response). Their stories suggest that gender norms strongly influence four key aspects of young people’s lives: mobility; sexual harassment and assault; education and labour; and relationships including marriage. Gender norms operating at the family, community, and broader social levels reflect traditional roles for women, shaping their daily experiences of limited mobility and street harassment, dictating their access to education and in turn shaping their role in the labour market, and ultimately directing them toward marriage and remaining at home.

### Mobility

In their stories, young women described their parents or older brothers restricting their movements. They had to seek permission to go out, even when accompanied by a chaperone, as in the following story:

Boys can sit with their friends as long as they want. But we can’t spend much time with our friends. If I want to go out to buy something, my mother doesn’t let me go. If I were a boy, I could have taken/brought anything of my choice. I can’t go out alone. My mother always goes with me, but I can’t take her everywhere. I can’t do anything by myself, I am dependent on others. (Session 9)

Participants described how in some families, brothers have the authority to restrict their sisters’ mobility. In the following excerpt, a young woman shared how her brother limited her mobility in response to neighbourhood gossip:

My brother has a friend, he sometimes lies to my brother that I saw your sister roaming around there and my brother believes his words. Then he stops me from going out, he also says to my mother that don’t let her go out of the house. (Session 11)

Participants noted that in addition to parents and brothers, community members also restrict their mobility. In one story, a participant recounted how a young woman in the neighbourhood became pregnant at age 15. Community members blamed the pregnancy on her freedom of movement and used it as a cautionary tale to place stricter limits on other young women in her community:

So people began to think that their children will also be spoil after watching all this. So they used to say that don’t let your children go out more. They have that old conservative mentality, they don’t allow girls to go out much, they don’t let them watch TV. They ask so many questions, where have you been? With whom you were talking on mobile? Why have you applied lipstick or lampblack? My parents don’t ask these kind of questions but other people ask such questions. My parents trust me but other people talk so much. (Session 7)

These stories demonstrate that pressure from the community further limits young women’s freedom even if their families do not.

### Sexual harassment and assault

The common practice of boys and men sexually harassing girls and women in public is also a barrier to their mobility. Several participants shared stories related to street harassment and assault, and reflected on how these experiences affected them. In one story, a young woman described being molested on her first trip to college in a tempo (autorickshaw):

There are certain advantages of being a woman but I think disadvantages are more. Because when we step out of our homes then people stare [at] us in such a manner that they have never seen a woman. When first time I was going to college, before that I had never come out of the house, I was sitting in a tempo, then there was a guy, he touched me at my back and I started crying. I cried and shouted, I yelled at him saying that take your hands off me and I jumped off the tempo. (Session 1)

In their stories, young women reacted in multiple ways to harassment, from feeling afraid and ashamed, to fighting back. One participant described an incident on the bus in which she confronted her attacker:

Then he took his bag in his hand and he started slowly touching me. First I thought this was just by mistake, I felt uncomfortable and the lady who sat next to me said that why don’t you sit comfortably, then he also said that be comfortable. Once we were all seated he started doing this again, but this I was better prepared, I pulled out my pen from my bag and jabbed/poked it deeply in his skin. Then he stood up, and I got proper place to sit. (Session 1)

Participants described how their families address the issue of harassment in a variety of ways. Some families sequestered their daughters or required chaperones when going outside of the home. In the example below, a participant shared how her mother encouraged her to resist harassment while still being cautious about how men could harm her.

Once I told my mother about it, she said no matter who is he; you should teach him a lesson.

So now I give them an answer. But sometimes we feel scared. That, they can do something wrong with us. They can do anything; they can throw acid on us. So, we feel a little scared. We should answer them but within a limit. We should be careful. (Session 7)

Experiencing harassment can also lead to confinement. In one story, a young woman recalled her brother cutting off her access to mobility and education after her harassers beat him:

Initially I didn’t share this with anybody at my home. But then I thought that this has been a regular practice, if I won’t tell my parents about it, then it would be wrong, so I should tell my family about this. So, I told my brother, then he went to school and asked him, why do you talk dirty to my sister. When my brother said all this, he started abusing my brother and they had a fight and my brother raised his hand, he called his friends from school and they all started beating my brother. After that my brother came home and told my parents that from now onwards she will not go to school. After that my school was stopped, I used to stay at home doing nothing. (Session 11)

In this instance, the family concluded that it was easier not to educate their daughter than to risk her being publicly harassed. In other instances, it was the victim who no longer wanted to leave home to go to school:

Once when I was ten years old, one day we two or three girls were going then some boys had teased us very badly. That incident was so scary that we were badly disturbed. When I reached home, I told my mother that I don’t want to go to school and I don’t want to study. After that when my mother used to insist me to go to school, I couldn’t tell her what had happened with me, what those boys did with us. (Session 3)

In both cases, the threat and experience of harassment limited girls’ mobility and access to education.

### Education and labour

As in the examples above, young women’s grip on education is tenuous, as many factors limit their access to schooling. Participants’ stories described how family attitudes and financial status hindered their educational goals. Educating young women was not uniformly prioritized in participants’ households. Whether and whom to send to school sometimes sparked conflict among parents and with extended family. In several cases, one parent championed the daughter’s right to education, with fathers often being the final decision maker. The following story provides a rare example in which a mother took on the decision maker role:

I had the urge to study. My father was alcoholic and he didn’t send us [to] school. I was very fond of studies, when I saw the other kids to go to school I also wanted to go to school. Then I decided that I have to study so I came to [school] with my sister and two three other girls. We enrolled here in [school] and we began to study. My father tried to stop us that if you will go to school then who will earn money, but my mother supported us, she fought with our father and she said that I will send them to school. They will also work and will do study too. (Session 1)

Some stories illustrate how education is a privilege reserved for those who do not need to work. In poor households, education is not only an additional expense, but also one that prevents youth from working and contributing to the household income. Another story illustrates the complex interplay of family finances, work, school, and cultural norms:

My father didn’t like that my mother or us (I and my sisters) going out to work, because he had that mentality that women should not go out of the house to earn. So we started a handicraft work (tie knot) at home. And for a complete year none of us were going to school and we all used to work. With that we collected some money, but my father’s opinion was that if someone in our family needs money then we have to help them, so when my uncle asked for money from my father, he gave him our money which we had collected by doing tie-knot work. This money was accumulated so that we could be enrolled to school, my mother was very clear that this money will be used only for admissions. That time we were very confused that what should we do, because schools were about to start and we didn’t have any money left. (Session 1)

In some stories, internal dynamics in the home (e.g. domestic violence, substance use) forced youth to work outside the home to earn money. In one story, a young woman’s alcoholic father beat her mother so badly that she could no longer work. The adolescent took a job and attended school to support her mother. In this case, her actions shifted the power dynamics in the family, prompting an end to the physical abuse:

My father used to drink and fight with us; sometimes he used to beat us. He still drinks and does the same things but now as we are studying he is afraid that if he will beat my mother then we will come forward to stop him. So now he can’t beat my mother, the one thing I don’t like about him is that he says that I have done everything for you, but that’s not right, because I work as a domestic helper. (Session 3)

Another young woman described balancing work and school responsibilities. In this story, getting an education allowed her to support her family financially:

I worked with my mother for three to four years. I was in class 9 by then I used to work as domestic help in three houses, apart from that I also worked at [name of NGO in Lucknow] as part time. I was interested in Math’s and Science, though I didn’t even know the meaning of Engineering but I always dreamed of becoming an engineer. But as they say sometimes what you want you don’t attain that. Unfortunately I was failed in 10th class; I had to change my subject, then I passed my 10th standard and came in 12th class… . Now Aunty has made me in charge of Uniform store. I fully support my mother now … there have been hurdles a lot of time but if you keep doing what you have to do and you have trust in god then everything will be fall in your hands. (Session 1)

Gender norms also influence who is allowed to work outside of the home and the type of work they do. Women stay at home and mainly clean and cook. If women must earn money outside the home, they typically clean, cook, and do household chores for others. One young woman mentioned starting a home business with her mother, i.e. handicraft work that could be done and distributed from their house, balancing financial needs with social norms.

Due to gender norms that limit many women to domestic work, which does not require formal schooling, the education of daughters is not always prioritized in families. Even when it is valued and desired for children, a family’s economic insecurity may prevent investments in education.

### Relationships and marriage

As marriage is of prime cultural importance to a family’s societal reputation and social mobility, family norms regarding marriage have a large influence on the gender norms that guide young women’s lives. The story circles revealed a number of these norms and their effect on young people. A woman must be chaste to be married. Young women and men may not associate with one another outside of marriage and their immediate family. This strict separation encompasses all social spheres, from outdoor play to school. Romantic relationships are prohibited by families and communities, leading young men and women to avoid overt friendships. Parents and brothers even use corporal punishment or other forms of abuse to prevent girls and women from interacting with boys and men. School is one of the few places where young women and men interact in a common space.

Gender norms regarding male-female interactions are reinforced harshly. One participant described being beaten by her mother and brother for playing with boys after school:

I was ten years old, I did not know the difference between a boy and girl. In the evening, it was the time of our coaching classes. After coaching all children were playing outside, I also started playing with them. Suddenly my mother came and saw me playing there. She took me home and then she had beaten me so badly. I didn’t even understand why she was beating me. After that she took me to my teacher then he explained to my mother that your daughter is not that type of girl, she is a good girl. Then my mother told my brother about this and he also beat me up. I was thinking why they are beating me, they have beaten me because I was playing or because I was lying that coaching class was over earlier and so we were playing there. But I wasn’t lying. My mother told me later that you were playing with boys that’s why I’ve beaten you. (Session 7)

In their stories, young women described the complex interplay between marriage and education. Local practices regarding marriage (e.g. who gets married, when they get married, and how much choice girls have in the matter) influence the status and perceived value of young women in society. Young women have little control in selecting potential partners; families decide when and whom they marry. In their stories, participants relay that their families send them to school until they get married or until they reach an appropriate age for marriage. For some, a certain school grade/standard signalled the end of their education, after which they would wait for marriage.

These stories suggest that education is a means to better marriage prospects for young women, rather than career ambitions. In contrast, participants expressed valuing learning more than finding a suitable partner. A few wanted to study or return to school to complete their education following marriage. Conflicting views on marriage and education within the family are highlighted in the following story:

When I was in 10th class then my father started looking for a groom for me. I wanted to study; suddenly one day there was a groom who came to my home to see me, I didn’t even know about this. And I said no in front of him that I don’t want to marry. I said in front of them that you give me in writing on stamp paper that you will let me study even after marriage. My father did not agree with this, he felt very humiliated, because in our society if a girl refused to marry then it doesn’t considered good. (Session 1)

Having either too little or too much education may make it more difficult for a young woman’s family to find a suitable match. In one story, the young person described her parents’ defiant reactions to their extended family’s traditional views on her education and marriage prospects:

Like I am doing higher studies, so my uncle, aunty and my neighbours say that you are educating your daughter so much, it will be difficult to find a good match for her. She is a girl so why are you educating her so much, in the end she has to do household chores. Don’t educate her so much, she is a girl, how will you arrange so much dowry for her wedding. They say so many things but my parents say that my daughter will study as much as she wants and I will not pay any dowry for her wedding. She can study as much as she wants. My parents did not listen to anyone. (Session 7)

Thus, young women who seek education are often criticized by their families and community, who consider educating young women to be unnecessary and a hindrance to marriage due to higher dowry.

## Discussion

Uttar Pradesh has some of the most pronounced gender disparities in education and employment in India, carrying health implications for girls and women. This study used story circles, a participatory research method, to collect personal narratives about young women’s lives in urban Uttar Pradesh, helping to map the mechanisms for these disparities. In response to prompts about daily challenges, their futures, and the role that gender plays in their lives, young women told stories of societal norms restricting their movement; experiences of harassment and assault; and familial, social, and economic challenges to furthering their education – all of which shape their future life directions and opportunities for employment, education, and marriage. According to participants’ stories, prevailing societal beliefs dictate that young women will eventually marry and work within the domestic sphere – their present opportunities are limited accordingly. Therefore, gender inequality and the culturally prescriptive attitudes and behaviours it encourages, is a pressing community health and development issue.

Fear of threats to young women’s ‘value’ (i.e. purity) for marriage by placing limits on their mobility, social interactions, and access to education is a priority for many families and has been documented in other studies conducted in India (Basu, Zuo, Lou, Acharya, & Lundgren, 2017). Gender norms create an ecology of factors that undermine young women’s ability to have alternative futures beyond those prescribed by their culture. Education is undervalued. In this study, some mothers supported their daughters in resisting norms, however, mothers are also undervalued and not always capable advocates for their daughter’s education. Even when a family holds progressive attitudes towards young women’s education employment, their views might still be countered by regressive community norms. These participants were recruited from urban slums, yet few families saw their education as a means to economic support or an end in of itself. Instead, it is seen through the lens of its impact on marriage.

In our study, lack of mobility, limitations on school attendance, and other cultural expectations based on gender converged to limit young women’s opportunities in the labour market to domestic employment trajectories. A case study conducted by Bose (2007) links restrictions on mobility to reduced labour market options for women. These restrictions limit women to taking jobs close to home, and coupled with little education, result in domestic and home-based work that is poorly compensated and encourages little empowerment. Gender segregation in occupations for women has been documented in India, emphasizing domestic and service jobs (Agrawal, 2016). These professions are also more concentrated among women of lower socioeconomic class and with less education, reinforcing the cycle of poverty through limited economic opportunity and low-paying work (Sarkar, 2015).

Gender norms also manifest through harassment and violence, daily realities for many young women in India. Eve teasing (street harassment) and sexual harassment have far-reaching consequences, including both short and long-term socio-emotional consequences and limited access to education for victims (Talboys et al., 2017). Participants’ stories highlighted harassment experienced on public transportation, which other studies have found to be commonplace (Dhillon & Bakaya, 2014; Gekoski, Gray, Adler, & Horvath, 2017; Leach & Sitaram, 2007; Tripathi, Borrion, & Belur, 2017). Given the widespread familial and social prohibitions limiting young women’s movement, harassment in public and on public transportation remains a significant barrier to young women’s education and employment opportunities, as is seen elsewhere in developing countries (Porter et al., 2011). Dhillon and Bakaya (2014) describe apathetic attitudes among law enforcement despite increasing reports of harassment in their study of women on public transportation in Delhi.

Economic barriers also limit young women’s access to education, despite national efforts by the Indian government to support their education, including scholarships for girls and financial incentives for families to keep their daughters in school (Sekher, 2010). A number of young women in this study balanced domestic responsibilities with their schooling, which helped justify their education. In the face of limited familial support, some young women were able to balance school and work. Schools that support young women through work-study or cash transfer programs, and/or schools that accommodate young women’s domestic or other work obligations through more flexible schedules, appear to meet their needs to balance household responsibilities with their own personal goals (Herz, 2002; Lavinas, 2001; Morley & Coady, 2003; Schultz, 2004). However, the potential success of government initiatives to support young women’s education is ultimately limited by prevailing cultural and familial norms that devalue their educational and economic empowerment, as well as rampant harassment and violence towards women and girls that restrict their mobility outside of the home.

### Strengths and limitations

This study used a novel and participatory method to explore a complex issue facing young women in India. Inherent to the methodology, by providing a broad prompt, participating storytellers are given the freedom to share a story of their choosing which might be either a positive or negative experience from their lives, providing young people with greater agency in the research process. This process allowed the participants to respond with stories and topics that mattered to them that were not limited by the researchers’ preconceived notions of young people or local culture.

Limitations to this study must be noted. This study relies on a small population of young people in Uttar Pradesh. While the findings are consistent with other studies, findings from this study are not generalizable to all young women in India. In addition, due to the participatory nature of the story circle methodology, the initial story would often set the tone for the following stories, leading to similar stories or stories on a shared theme. While consistency builds confidence that the stories have identified a common experience of young women, the methodology may limit the breath of comments seen in methodologies such as focus groups.

## Conclusion and implications

Story circles offer an innovative method for learning about the multiple ways in which young women experience the world and come to understand their place in it given their gender. Use of participatory research methods, such as story circles, enables youth to share information about themselves and communicate about their lives with contextual complexity. Participants’ narratives revealed that mobility restrictions, rampant sexual harassment and assault in their communities, limited educational and economic opportunities, and the dominant belief that marriage is the utmost important goal for young women all intersect as mechanisms of gender inequality.

Findings from this study suggest that India’s persistent gender inequality will require multi-pronged solutions and interventions addressing social, structural, educational, and economic factors. Measures to transform familial and broader cultural attitudes around women and girls’ potential for academic and career achievement should be considered. Allowing and supporting girls and women to be outside the home, free of harassment and violence, might increase their access to educational and economic opportunities. Broadening the flexibility of school schedules and recommitting to young women’s access and right to education, through family and community-level interventions, can be steps to support girls in realizing their educational and career potential, and better prepare them to participate in the economy. Finally, reinforcing the value of girls and women – in their own right and not simply for marriage, childbearing, and work in the domestic sphere – may help improve the overall health and wellbeing of women and by extension their families, surrounding communities, and the broader population.
